# Is It Time to Reassess the Target Age of Human Papillomavirus Vaccination Globally?

**DOI:** 10.1093/cid/ciaf679

**Published:** 2025-12-09

**Authors:** Edie Brassington, Cassandra Fairhead, Andrew Hill

**Affiliations:** School of Public Health, Imperial College London, London, United Kingdom; Acute Medicine, Royal Free London NHS Foundation Trust, London, United Kingdom; Department of Pharmacology and Therapeutics, University of Liverpool, Liverpool, United Kingdom

**Keywords:** human papillomavirus, vaccines, immunogenicity, cervical cancer, vaccination coverage

## Abstract

In 2022, 464 000 people died from human papillomavirus (HPV)–related cancer. Despite the availability of safe, highly effective HPV vaccinations, global coverage is just 20% for girls and 7% for boys. We summarized the evidence for target vaccination age and found that vaccinating at age 9–10 years, the youngest end of the World Health Organization's (WHO) recommended age range, has multiple advantages. Earlier vaccination increases the likelihood of vaccinating before HPV exposure, may reduce the impact of sex-associated stigma, and is logistically advantageous through enabling primary-school immunization and program resilience. Furthermore, we systematically reviewed published immunogenicity data on children aged 9–15 years and identified that vaccination at age 9 is immunologically robust. Globally, most countries target ages 11 years or older for HPV vaccination. Our findings indicate an urgent need to target earlier ages to help achieve the WHO’s goal to vaccinate 90% of girls against HPV by age 15.

## Teaser

Most countries target HPV vaccination at age 11 years or older. We summarized the evidence on vaccination age and found that vaccinating at age 9–10 years is immunologically robust and offers an opportunity to improve uptake globally.

**(See the Editorial Commentary by Kane and Franco on pages e884–6.)**

In 2022, 350 000 women worldwide died from cervical cancer and 660 000 new cases were diagnosed [1]. Cervical cancer is caused by persistent infection with high-risk human papillomavirus (HPV), with more than 70% of cases attributable to genotypes HPV16 and HPV18 [2]. Other HPV-attributable cancers include anal, oropharyngeal, penile, vaginal, and vulvar cancer. These contribute to a substantial health burden, causing over 114 000 deaths in 2022 [3]. In total, 5% of all cancers worldwide are estimated to be attributed to HPV [4]. Yet, safe and highly effective vaccinations for HPV have been available since 2006. So far, 150 countries report implementing at least a partial national vaccination program [5]. In countries with high vaccine coverage, the prevalence of vaccine-type HPV has declined by 73%–85% along with a 41%–57% reduction in high-grade cervical lesions over 10 years of implementation [6]. With the addition of screening and treatment for precursor lesions, cervical cancer is almost entirely preventable [1]. However, global vaccine coverage remains a sobering issue. In 2023, only 20% of females and just 7% of males worldwide received at least 1 dose by age 15 years [7]. This figure falls alarmingly below the World Health Organization’s (WHO’s) HPV cervical cancer elimination target of 90% for women and starkly contrasts with global coverage of other immunizations. In the same year, 83% coverage was achieved for full hepatitis B vaccination, which also has a role in cancer prevention [7].

Currently, the WHO recommends routine HPV vaccination for girls and boys aged 9–14 years [1]. One proposal to improve vaccination coverage is to vaccinate girls and boys as early as possible. Original registration studies included boys and girls aged 9 years and older, and the minimum age stated in Food and Drug Administration (FDA) and European Medicines Agency (EMA) guidelines is 9 years of age [8, 9]. In reviewing the available evidence, benefits of earlier vaccination, targeting ages 9 to 10 rather than 11 to 14, include the following: (1) a greater chance of vaccinating prior exposure to HPV, (2) potential for a reduction in the impact of sex-associated stigma, (3) logistical advantages in vaccinating children aged 9 years, and (4) robust immunogenicity and efficacy.

## A GREATER CHANCE OF VACCINATING PRIOR TO EXPOSURE TO HPV

Human papillomavirus is a globally prevalent sexually transmitted infection, and its transmission risk is closely linked to the age of first sexual activity [10]. The age of sexual debut varies both internationally and within countries, influenced by a complex interaction of sociocultural factors [11]. “Early sexual initiation,” defined as first intercourse before age 15, is reported across all WHO regions. A meta-analysis found that, worldwide, 14.2% (95% CI, 12.1%–16.2%) of adolescents aged 12–15 years had early sexual initiation, ranging from 19.5% (13.5%–25.2%) in high-income countries (HICs) to 7.3% (5.5%–9.0%) in low- or middle-income countries (LMICs) [11]. Earlier vaccination enables more individuals to be vaccinated prophylactically, increasing vaccination effectiveness [12].

Nonsexual (and nonperinatal) transmission routes, such as auto-inoculation and nonsexual skin-to-genital-skin contact, have been documented [13]. High-risk HPV seroprevalence has been observed in children under 15 years across multiple continents [14–17], suggesting that exposure is occurring earlier than previously recognized and is not solely linked to sexual debut. Several trials of HPV vaccination have identified baseline seropositivity to HPV in children aged between 9 and 14 years (see Appendix B, Supplementary Table 1). For example, a large multicountry trial reported immunoglobulin G (IgG) baseline seropositivity rates of 4.2% and 12% for HPV16 and HPV18, respectively, in children aged 9 to 12 across 17 countries [18]. Restrepo et al [18] only presented baseline seropositivity by HPV type from the per-protocol included (PPI) population. To be included in the PPI population, individuals had to be competitive Luminex immunoassay (cLIA) seronegative on day 1 (seropositivity cutoffs: ≥20 mMU/mL for HPV16 and ≥24 mMU/mL for HPV18). As a result, these figures likely underestimate baseline seropositivity to HPV compared with the wider population. In total, 8.5% and 5.6% of girls and boys, respectively, were cLIA seropositive to any of the 9 HPV types on day 1 (HPV 6/11/16/18/31/33/45/52/58) and were therefore excluded from the PPI population [19]. Similarly, a cross-sectional study in Mexico City including unvaccinated boys aged 9 to 14 years (mean age, 10.4 years) found substantial seropositivity using a reference laboratory standard, to HPV18, both in isolation (22%) and in combination with other serotypes (31%) [14]. When a more conservative cutoff was then used, to account for uncertainty in the optimum cutoffs, 15.2% still had evidence of contact with 1 or more HPV serotypes (HPV 6,11,16,18) and 3.5% of young boys had evidence of solely HPV18 exposure. Given that the target age of vaccination in Mexico is 11 years, these findings suggest that a proportion of individuals may be exposed to HPV prior to vaccination. However, interpreting baseline seropositivity is challenging due to undetermined cutoffs, the use of different assays, and a lack of evidence regarding prior exposure, persistent infection, and subsequent cancer risk. Nonetheless, this supports an epidemiological benefit to vaccinating as early as safety data allow.

## EARLIER VACCINATION IS AN OPPORTUNITY TO REDUCE SEXUAL STIGMA

Misinformation and stigma surrounding the HPV vaccine, especially surrounding sexual promiscuity, are a significant barrier to vaccination uptake globally [20, 21]. Table 1 summarizes attitudes towards HPV vaccination from studies in different global settings [22–29]. Evidence, predominantly from North America, has found that introducing the vaccine at age 9 reduced the incidence of sex-associated stigma in parents [30]. The vaccine was seen as a pediatric anticancer vaccine, rather than a marker of approaching sexual debut. Although evidence directly linking earlier vaccination to reduced stigma is limited, substantial research indicates that sexual stigma is a widespread and cross-cultural barrier to HPV vaccination (Table 1). Earlier vaccination could help to disentangle the association between HPV vaccination and sexual debut, reframing HPV vaccination as a childhood health measure, which has the potential to increase uptake globally.

**Table 1.** Perceptions of HPV Vaccination Associated With Sexual Stigma in Diverse Global Settings

Country/Region [Ref]	Year	Study Type^a^	Themes Identified
Hong Kong [22]	2017	Qualitative cross-sectional survey of adolescent girls aged 12–18 y and their mothers; n = 340	“Vaccinating for HPV will cause a girl to be stigmatized as promiscuous.” Themes identified: Fear of sexual promiscuity Encouragement of early sexual debut Perception that girls are too young to receive a vaccine for a sexually transmitted infection Concern that girls would be stigmatized if they accepted the vaccine Concern regarding side effects
Syria [23]	2018	Qualitative cross-sectional survey of Syrian mothers of schoolgirls in Aleppo City; n = 400	Themes identified: Daughters are not at risk due to mode of transmission Perception that girls are too young to be vaccinated against sexually transmitted infections The main barrier identified to vaccinate was high cost and lack of knowledge, but 2 themes were identified relating to sexual stigma/social norms.
India [24 ]	2019	Qualitative cross-sectional survey study of parents of school going adolescent daughters; n = 1609	“HPV vaccination will make girls sexually active.” Themes identified: Fear of sexual promiscuity Encouragement of early sexual debut
Kenya [25]	2021	Qualitative cross-sectional survey using a standardized questionnaire to mothers of adolescent girls; n = 300	“Vaccination of my daughters will prompt early sexual activity.” Themes identified: Holding a belief that HPV vaccination would lead to early sexual activity significantly reduced intent to vaccinate; however, predominantly positive attitudes towards HPV vaccination were identified
Multiple countries^b^ [26]	2021	Quantitative, cross-sectional survey study; n = 258	Themes identified: Feminine honor was an important cultural factor affecting intent to vaccinate daughters, when combined with a belief that vaccination encouraged sexual promiscuity; women higher in feminine honor endorsement were less likely to support vaccinating daughters
USA [27]	2023	Qualitative cross-sectional survey of parents with unvaccinated children of an eligible age (11–17 y); n = 636	Themes identified: Encouragement of sexual debut Promotes sexual promiscuity Perception that their child is too young to receive a vaccine for a sexually transmitted infection Children are not at risk due to mode of transmission
USA [28]	2023	Quantitative cross-sectional survey in unvaccinated women in university; n = 182	Themes identified: Feminine honor: a gendered code that emphasizes values, such as modesty and propriety; higher “feminine honor” endorsement was linked with higher levels of HPV stigma, shame, and embarrassment, decreasing HPV vaccination intent
England [29]	2024	Semistructured interviews of vaccine-hesitant, ethnically diverse parents in London; n = 29	“They’re teenagers, they’re strong, and so what are they protected against, I don’t understand.” “It wasn’t like she was going out and at risk of sexual assault and it wasn’t like she was choosing to have a relationship, she wasn’t partying, she wasn’t getting drunk. She wasn’t in any of those social situations.” Themes identified: Perception that adolescent vaccinations are less important than childhood vaccinations Perception that girls are too young to be at risk of HPV infection Perception that vaccination condones sexual activity Perception that girls are not at risk of sexual transmission of HPV Presence of taboo, judgment, and stigma

Abbreviations: HPV, human papillomavirus; Ref, reference.

^a^n = number enrolled.

^b^Online environment using Amazon Mechanical Turk (MTurk)—participant ethnicities: White (67.8%), Asian (13.3%), African American (10.5%), Latino/Hispanic (5.2%), or other (3.0%) [data on the participants’ country of residence was not available].

## LOGISTICAL ADVANTAGES IN VACCINATING CHILDREN AGED 9 YEARS

The optimal HPV vaccination strategy depends on compatibility with existing infrastructure, affordability, and capacity to achieve the highest possible coverage. School-based immunization programs have improved uptake globally [31]. Given the higher global enrollment rates in primary school (89% of girls and 91% of boys) compared with secondary schools (84% of both girls and boys), primary school vaccination presents an opportunity to increase coverage and simultaneously address HPV-related health inequities, by including children who do not progress to secondary education [32]. This strategy also allows admission to secondary school to serve as a safety net, to check immunization status and re-offer vaccination where indicated.

Irrespective of delivery strategy, vaccinating at age 9 enables resilience in immunization programs, particularly to unpredictable disruptions. The coronavirus disease 2019 (COVID-19) pandemic, for example, caused major disruptions to vaccination programs, especially in LMICs—Malawi's coverage fell from 83% to 13% [33]. Although vaccination paused, HPV exposure continued. More recently, reductions in global health funding, including US Agency for International Development (USAID) contributions to Gavi, threaten vaccine coverage internationally. If a child misses immunization, targeting vaccination at age 9 provides a larger window for intervention before the WHO target age of 15. A retrospective study found that those initiating vaccination at ages 9–10 years were 22 times more likely to complete a 2-dose schedule by age 15 compared with those starting at ages 11 or 12 years [34]. The increasing adoption of single-dose HPV schedules (now implemented in >60 countries) [5] will further simplify both school- and community-based programs, and facilitate timely vaccination before age 15, when the vaccine is most effective.

## EVIDENCE OF ROBUST IMMUNOGENICITY

There may also be an immunological advantage of earlier vaccination. A recent systematic review identified that younger individuals aged 9 to 14 years experienced a higher antibody response when compared with older adolescents and women aged 15–18 years [12]. However, to our knowledge, no identified review has so far analyzed the difference in immune response between ages 9 and 14 years. We aimed to bridge this gap by conducting a systematic review of the impact of age (within 9–14 years) on immunogenicity of the HPV vaccination.

**Figure 1.**
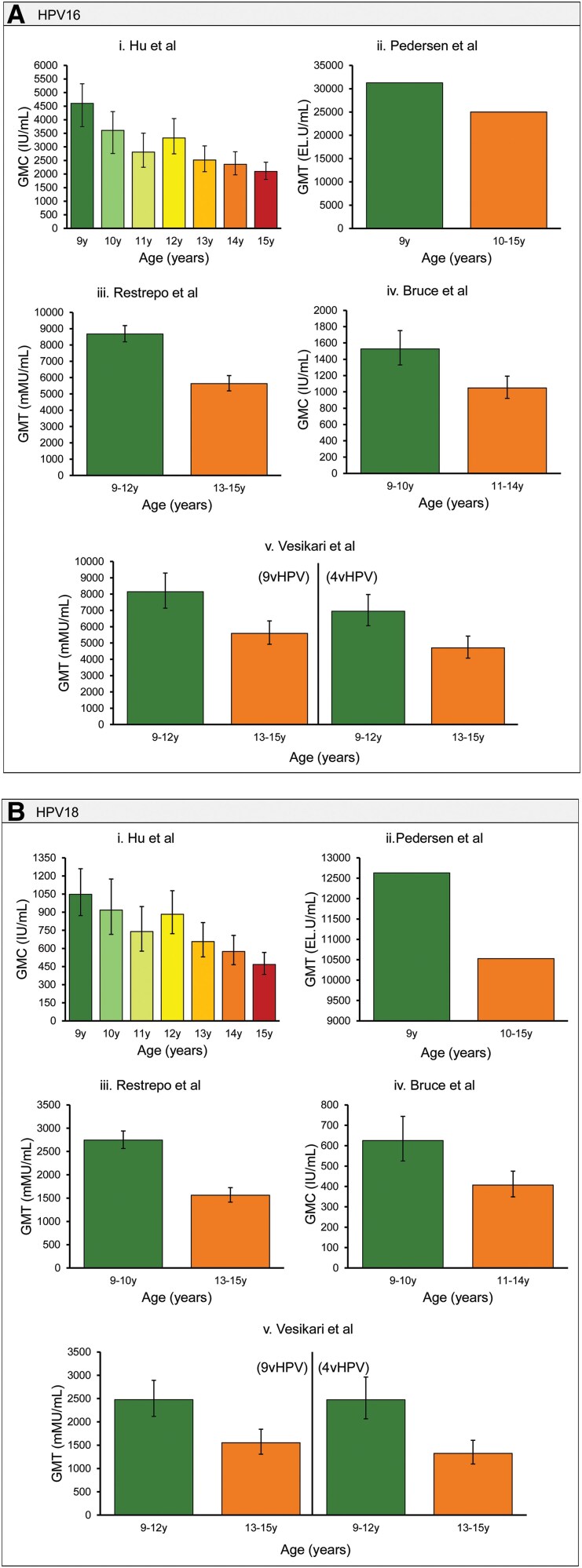
Geometric mean concentration (GMC)/geometric mean titers (GMTs) of HPV16/18 antibodies at month 7 following a 3-dose schedule, by age at vaccination. *A*, GMT/GMC of antibodies against HPV16; *B*, GMT/GMCs of antibodies against HPV18. Each bar graph represents a single study; colors distinguish between age groups. Abbreviation: HPV, human papillomavirus.

Medline and EMBASE (via Ovid) were searched on 4 March 2023 to identify articles that analyzed the immunogenicity of HPV prophylactic vaccinations. The search strategy contained MeSH (Medical Subject Heading) terms including “Papillomavirus Vaccines/” “Immunogenicity, Vaccine/” and “Seroconversion/” (see Supplementary Appendix A for the full search strategy). Studies were included if at least 1 presentation of either geometric mean titer/concentrations (GMT/GMCs) or seropositivity were stratified by age and schedule of vaccination. Of the 3102 articles in our initial search, 8 studies provided appropriate age-disaggregated data (Table 2). Only data for HPV16 and HPV18 genotypes were analyzed as these account for 70% of cervical cancers [2]. Seven studies assessed 3-dose vaccination schedules, 3 evaluated 2-dose schedules, and 1 study reported interim immunogenicity data following a single dose administered at day 1 of a 2-dose (vaccination at month 0 [day 1] and month 12) schedule. We found that, in the 9–14-year age group, vaccinating the youngest children was immunologically robust (Figure 1; Appendix B, Supplementary Table 2). It may result in a higher magnitude of immune response, as indicated by non–statistically significant trends of GMT/GMCs and seropositivity. However, in no studies were these trends statistically significant, and as very low levels of antibody are protective, small differences in antibody titers are likely not clinically significant.

**Table 2.** Characteristics of Studies Analyzing the Immunogenicity of HPV Prophylactic Vaccinations, Identified Through Systematic Review

Study [Ref]; Trial ID	Year	No. of Participants	Country	Study Design	Vaccine and Dose (D)^a^	Sex	Age Group, y	Follow-up, m
Reisinger et al [35 ]; NCT00092547	2006	1781	International^b^	Randomized, double-blind, placebo-controlled, parallel trial	4-valent 3D (02,6)	M + F	9–12, 13–15	18
Pedersen et al [36]; NCT00578227	2011	814	Canada, Denmark, Hungary, Sweden	Randomized, open, controlled study	2-valent 3D (02,6)	F	9, 10–15	7
Vesikari et al [37]; NCT01304498	2015	600	Belgium, Denmark, Finland, Italy, Spain, Sweden	Randomized, double-blind, controlled study	9-valent 4-valent 3D (02,6)	F	9–12, 13–15	7
Hu et al [38]; NCT02562508	2019	979	China	Randomized, age-stratified, immuno-bridging study	2-valent 2D (0,6) 3D (02,6)	F	9, 10, 11, 12, 13, 14, 15	30
Yao et al^c^ [39]; NCT03206255	2022	875						
Sauvageau et al [40]; NCT00501137	2019	253	Canada	Randomized, age-stratified, noninferiority immunogenicity study	4-valent 2D (0,6)	F	9, 10, 11, 12, 13	24
Bruce et al^d^ [41]; N/A	2020	525	USA (Alaska)	Prospective cohort study	4-valent 3D (02,6)	M + F	9–11, 12–14	24
Bornstein et al [42]; NCT01984697	2021	1518	International^e^	Randomized, open-label, noninferiority trial	9-valent 2D (0,6), (0,12)	M + F	9–10, 11–12, 13–14,	36
Restrepo et al [18]; NCT00943722	2023	3074	International^f^	Randomized immunogenicity study	9-valent 3D (02,6)	M + F	9–12,13–15	126

Abbreviations: F, female; HPV, human papillomavirus; M, male; N/A, not available; Ref, reference.

^a^(02,6) = vaccination at month 0 (day 1), month 2 and month 6; (0,6) = vaccination at month 0 (day 1) and month 6.

^b^Ten countries in North America, Latin America, Europe, and Asia.

^c^Yao et al is the follow-up study for Hu et al, although it has a different trial identifier number.

^d^The study conducted by Bruce et al had no identifiable NCT number.

^e^Canada, Chile, Colombia, Czech Republic, Denmark, Israel, Malaysia, Norway, South Africa, South Korea, Spain, Taiwan, Thailand, Turkey, United States.

^f^Austria, Belgium, Brazil, Chile, Colombia, Costa Rica, Finland, India, Peru, Poland, South Africa, South Korea, Spain, Sweden, Taiwan, Thailand, United States.

### GMT/GMC Responses to HPV Vaccination With Age

Figure 1 presents GMT/GMC data from 5 studies, measured at month 7 after a 3-dose schedule and stratified by age of vaccination. Month 7 is a commonly used marker of immune response for HPV as it signifies 1 month after the last dose of vaccine. In 1 study [35], age comparisons were described narratively, but no raw data were provided; therefore, these findings could not be presented graphically. As age of vaccination increased, the magnitude of antibody response decreased, although no studies reported statistical significance. In 3 high-quality studies [18, 35, 37] comprising 3926 participants, children aged 9 to 12 years exhibited 1.5-fold greater GMTs for HPV16 and 1.6- to 1.8-fold greater GMTs for HPV18 than adolescents vaccinated at 13 to 15 years (Figure 1; Reisinger et al [35] data are not shown graphically). Although fewer studies presented data for a 2-dose regimen, trends were similar (Appendix B, Supplementary Figure 1). The exception to this trend was Sauvageau et al [40], who reported a peak immune response in the 11–12-year age group, although, again, the differences between age groups were not statistically significant. The interpretation of this finding is limited by the study's small sample size, particularly the very small subgroup of 9-year-olds (n = 18). One study included in our review provided data from a single dose of vaccination. Antibody responses to HPV16 and HPV18 peaked in girls vaccinated at ages 11–12, with a noticeable decline in immunogenicity in girls vaccinated after age 12. In boys, no distinct peak was observed, but a gradual age-related decrease in antibody levels was noted. Again, these trends did not meet statistical significance (Appendix B, Supplementary Figure 2). The mechanism underlying this difference warrants further investigation. Yang et al [43] found a similar trend with severe acute respiratory syndrome coronavirus 2 (SARS-CoV-2) vaccination, finding that younger children exhibit higher levels of IgG than adolescents and young adults, although similarly, the mechanisms have so far remained unclear. Nevertheless, these data support the initiation of vaccination at age 9 years.

### Long-term Immunogenicity

**Figure 2.**
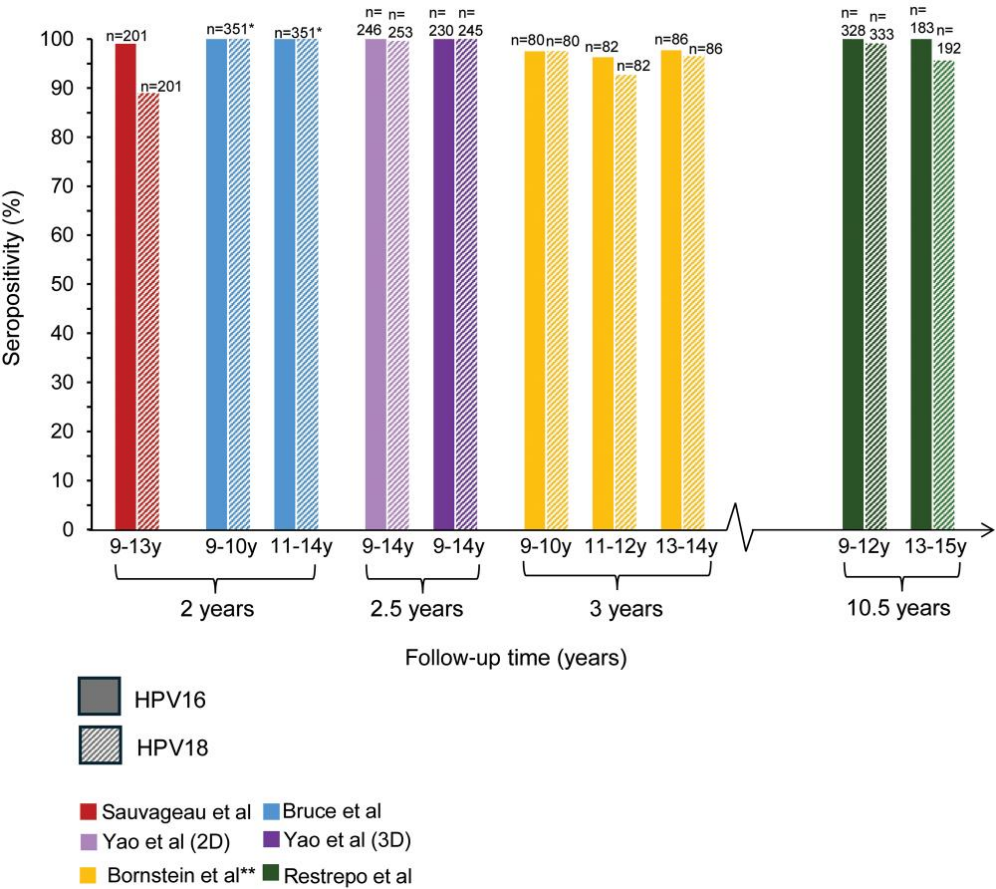
Proportion of participants who remained seropositive for HPV16 and HPV18 at 2, 2.5, 3, and 10.5 years after completion of the primary vaccine series. Individual studies are colored and stratified by age at first dose. Seropositivity was defined according to each study's validated assay cutoff. Numbers above the bars indicate total participants assessed at each time point. For graphical representation purposes, HPV16 seropositivity at 2 years postvaccination, as reported by Sauvageau et al [40], is depicted as 99.5%. The actual reported range is 99%–100%. *n = 351: Bruce et al [41] did not provide the number of participants disaggregated by age group; therefore, the total number of participants was used instead. **For clarity purposes, only the seropositivity of females in Bornstein et al [42] is represented graphically. Both male and female seropositivity values are presented in Appendix B, Supplementary Table 2, and follow a similar pattern. Abbreviation: HPV, human papillomavirus.

An initial concern regarding early vaccination was that immunogenicity might wane before sexual debut or during periods of highest sexual risk. However, up to 10.5 years of follow-up data are now available, demonstrating a robust and sustained immune response. We assessed the longevity of immune response in 7 of 8 studies with available age-disaggregated data. Across all studies, in participants aged 9–14 years, seropositivity and antibody titers declined over time. However, younger individuals maintained similar or numerically higher levels compared with older participants (see Appendix B, Supplementary Table 2). Figure 2 demonstrates sustained seropositivity to HPV16 and HPV18 at 2, 2.5, 3, and 10.5 years postvaccination. These findings are reassuring with respect to longevity of response and suggest that earlier vaccination provides durable protection. Restrepo et al [18] presented seropositivity rates detected by 2 assays: IgG-LIA and cLIA. Given the absence of an internationally established immunological correlate of protection for infection, we present the IgG-LIA seropositivity results graphically. Seropositivity rates were slightly lower when measured by cLIA (presented in Appendix B, Supplementary Table 2), but both assays demonstrated high proportions of seropositivity. Using data from the cLIA, if an individual is vaccinated at 9 years of age, 98.2% and 84.4% are expected to remain seropositive to HPV16 and HPV18, respectively, to at least 19 years of age, with no evidence of further decline. Data from the IgG-LIA suggest a higher rate remain seropositive, with 100% and 99.1% of 9- to 12-year-olds seropositive to HPV16 and HPV18, respectively, at 10.5 years of follow-up. The absence of a universally agreed-upon GMT/GMC or seropositivity correlate for immune protection against HPV is a limitation of this evidence. Nonetheless, the HPV vaccines are highly immunogenic [2].

### Limitations

Recognizing the efficacy and logistical advantages of single-dose vaccination, the WHO updated its recommendations to a 1-dose schedule in September 2023. However, data comparing immunogenicity between ages 9 to 14 years in this recently introduced regimen remain limited. Only 1 study included in our systematic review provided age-disaggregated data for a 1-dose schedule [42]. Although a similar trend as 3- and 2-dose schedules was observed (Appendix B, Supplementary Figure 2), further investigation would be helpful to confirm the impact of age on immunogenicity in 1-dose regimens. Currently, the evidence is reassuring. A PATH technical synthesis on single-dose vaccination reported a durable immune response, noting that stronger responses are observed in preadolescent boys and girls (aged 9–11 years) compared with older ages [44]. Furthermore, long-term immunogenicity data will continue to emerge from multiple trials, including the KENya Single-dose HPV-vaccine Efficacy and the Dose Reduction Immunobridging and Safety Study of two HPV vaccines in Tanzanian girls trial [44]. The availability of age-disaggregated data from these studies will be key in addressing the current evidence gap.

No studies that met the inclusion criteria for our systematic review included participants with human immunodeficiency virus (HIV). Yet, this population is critically important to consider, as women with HIV are 6 times more likely to develop cervical cancer than women without HIV [1]. Future research evaluating the immunogenicity of vaccination within this population is essential. Current evidence is reassuring; Levin et al [45] found that the quadrivalent HPV vaccine elicited seropositivity rates exceeding 96% in children with HIV as young as 7 years of age.

### Current Global Approaches to Age of HPV Vaccination

**Figure 3.**
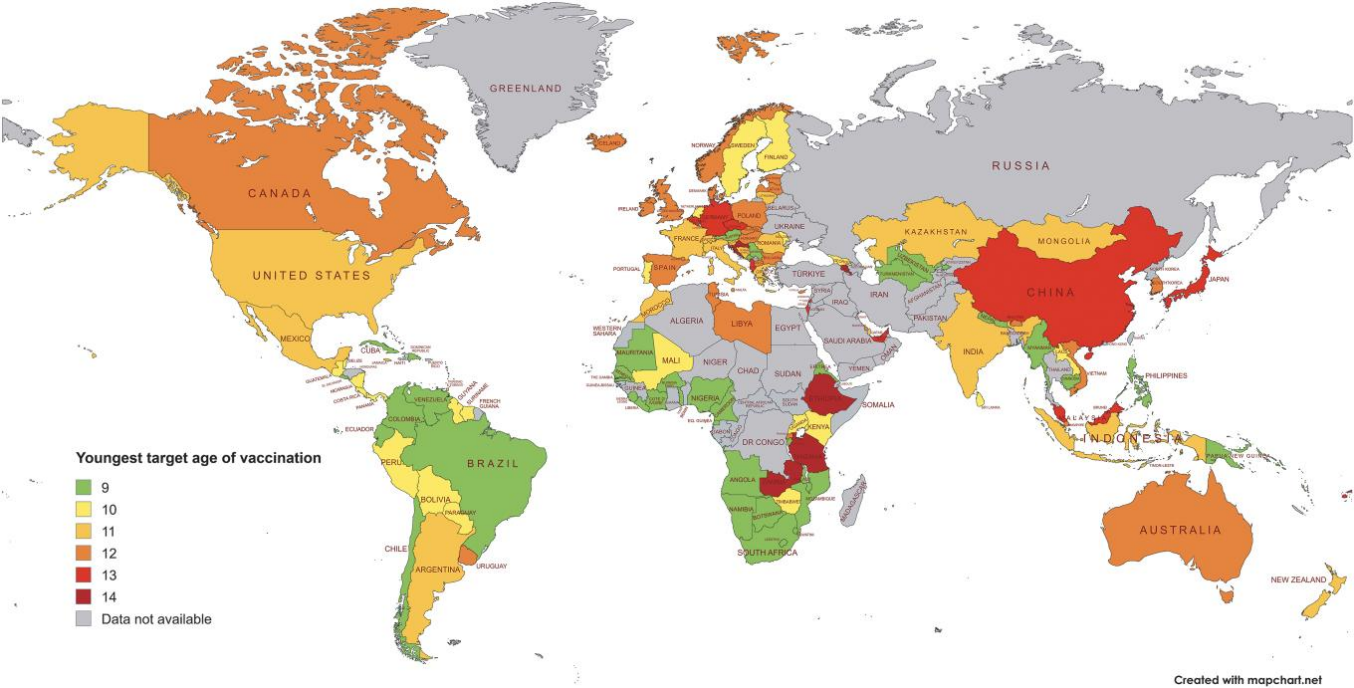
Map showing the global distribution of the youngest target age of vaccination by country. Most vaccination program target ages were obtained from the World Health Organization. A comprehensive table of references is provided in Appendix B, Supplementary Table 5.

Despite the inclusion of ages 9 and 10 within WHO's recommendation, globally most countries target the upper part of the age range. Currently, 52% of countries initiate vaccination at age 11 years or older (Figure 3). We mapped global HPV vaccination target ages, adopting a “best-case scenario” approach by assuming that countries targeting multi-age cohorts aim to reach the youngest age within the range. Based on this method, only 30% of countries target, or plan to target, children aged 9 years. A full summary of the target age of vaccination by country and income classification is provided in Appendix B, Supplementary Table 3. High-income countries tend to target older adolescents: 78% of HICs initiate vaccination at age 11 years or above, compared with 54% of LMICs. Generally, newer programs target younger age groups, whereas older programs target older adolescents (Appendix B, Supplementary Table 4). This may reflect the fact that earlier programs were designed prior to the availability of data supporting a long-term immune response. The emerging evidence on safety, long-term immunogenicity, and the uptake benefits of earlier vaccination highlights the need for older programs to adapt their target age to younger individuals.

## CONCLUSION

Globally, only 1 in 8 girls and 1 in 25 boys are vaccinated against HPV despite the availability of effective vaccines since 2006 [7, 46]. Consequently, the burden of HPV remains significant, with over 920 000 cancer cases and 464 000 associated deaths reported in 2022 alone. Vaccinating children at 9 years of age is immunologically robust and offers an opportunity to strengthen vaccination programs. It increases the likelihood of vaccinating prior to HPV exposure, may reduce sex-associated stigma, provides a longer window to catch up if vaccination is missed, and has logistical advantages in reaching primary-school-aged children. This strategy has the potential to improve vaccine uptake globally and ultimately reduce the incidence of these preventable cancers.

## Supplementary Material

ciaf679_Supplementary_Data

## Supplementary Data

Supplementary materials are available at *Clinical Infectious Diseases* online. Consisting of data provided by the authors to benefit the reader, the posted materials are not copyedited and are the sole responsibility of the authors, so questions or comments should be addressed to the corresponding author.
