# Supplementary material for: Is It Time to Reassess the Target Age of Human Papillomavirus Vaccination Globally?
Source: Clin Infect Dis. 2025 Dec 9;82(4):e875–83. doi: 10.1093/cid/ciaf679 (PMC13131925; doi:10.1093/cid/ciaf679)
Supplement: ciaf679_Supplementary_Data [file ciaf679_supplementary_data.docx]

**Appendix A:**

OVID and EMBASE final searches:

**Ovid MEDLINE(R) ALL <1946 to March 4, 2024>**

1 exp Papillomavirus Vaccines/ or HPV Vaccin*.mp. 14213

2 Papillomavirus vaccin*.mp. 11336

3 Gardasil.mp. or exp Human Papillomavirus Recombinant Vaccine Quadrivalent, Types 6, 11, 16, 18/ 1139

4 Cervarix.mp. 356

5 HPV L1 VLP vaccin*.mp. [mp=title, book title, abstract, original title, name of substance word, subject heading word, floating sub-heading word, keyword heading word, organism supplementary concept word, protocol supplementary concept word, rare disease supplementary concept word, unique identifier, synonyms, population supplementary concept word, anatomy supplementary concept word] 14

6 (HPV adj1 Vaccin*).mp. [mp=title, book title, abstract, original title, name of substance word, subject heading word, floating sub-heading word, keyword heading word, organism supplementary concept word, protocol supplementary concept word, rare disease supplementary concept word, unique identifier, synonyms, population supplementary concept word, anatomy supplementary concept word] 11010

7 1 or 2 or 3 or 4 or 5 or 6 14791

8 Immunogenicity, Vaccine/ 3443

9 Immunogenicity.mp. 52105

10 Immunoglobulins.mp. or Immunoglobulins/ 86692

11 exp Antibodies, Viral/ or Seropositivity.mp. 158853

12 Seroconversion/ 1214

13 ELISA assay.mp. or exp Enzyme-Linked Immunosorbent Assay/ 159996

14 exp Immunoassay/ or cLIA.mp. 503855

15 8 or 9 or 10 or 11 or 12 or 13 or 14 758745

16 7 and 15 1239

17 limit 16 to yr="2001 -Current" 1213

**EMBASE SEARCH:**

Embase Classic+Embase <1947 to 2024 March 12>

1 HPV Vaccin*.mp. 14511

2 exp Human papilloma virus vaccine/ 3084

3 Papillomavirus vaccin*.mp. 4751

4 Gardasil.mp. 2952

5 Cervarix.mp. 2084

6 HPV L1 VLP Vaccin*.mp. 14

7 (HPV adj1 Vaccin*).mp. [mp=title, abstract, heading word, drug trade name, original title, device manufacturer, drug manufacturer, device trade name, keyword heading word, floating subheading word, candidate term word] 14677

8 1 or 2 or 3 or 4 or 5 or 6 or 7 18638

9 Immunogenicity, Vaccine.mp. or exp vaccine immunogenicity/ 7716

10 Immunogenicity.mp. 108389

11 seropositivity.mp. 21349

12 exp virus antibody/ 113608

13 seroconversion/ 30574

14 exp enzyme linked immunosorbent assay/ or ELISA assay.mp. 503620

15 exp enzyme linked immunosorbent assay/ or ELISA assay.mp. 503620

16 CLIA.mp. 6023

17 exp immunoassay/ 783206

18 9 or 10 or 11 or 12 or 13 or 14 or 15 or 16 or 17 993143

19 8 and 18 1921

20 limit 19 to yr="2001 -Current" 1889

**Appendix B:**

**Supplementary Table 1.** Baseline HPV-16 and HPV-18 seropositivity rates at enrolment in HPV vaccine trials. (*M = male, F = female)*

|  | **Baseline Seropositive on enrolment (%)** | | | | | | |
| --- | --- | --- | --- | --- | --- | --- | --- |
|  | ***Sex*** | ***Age range*** | ***HPV 16*** | ***HPV 18*** | ***n*** | ***Assay type*** | ***Laboratory reference*** |
| *Pedersen et al* | *F* | *9-15* | *7.0* | *3.5* | *542* | *ELISA* | *HPV16: 8 EL.U/mL*  *HPV18: 7 EL.U/mL* |
| *Hu et al* | *F* | *9-14*  *(IgG antibody)* | *11.9* | *7.9* | *604* | *ELISA (for IgG)* | *ELISA:*  *HPV16: 3.0IU mL-1 HPV18: 2.1IU/mL-1* |
|  |  | *9-14*  *(Neutralizing)* | *0.5* | *1.0* | *604* | *PBNA (for neutralizing)* | *PBNA:*  *HPV16: 2.1IUmL-1*  *HPV18:1.7IUmL-1* |
| *Dobson et al ^1 *^* | *F* | *9-13* | *0* | *0* | *253* | *cLIA* | *HPV16: ≥20mMU/mL*  *HPV18: ≥24 mMU/mL* |
| *Iversen et al ^2 **^* | *M + F* | *9-14* | *1.7* | *1.5* | *1204* | *cLIA* | *Not specified* |
| *Restrepo et al **** | *M+F* | *9-12* | *4.2* | *12* | *358* | *IgG-LIA* | *HPV16: ≥7mMU/mL*  *HPV18: ≥10mMU/mL* |
|  |  | *13-15* | *6.6* | *8.4* | *167* |  |  |

^* The baseline seropositivity data for the study referred to in Sauvageau et al (included in our systematic review), was available from Dobson et al. ** The baseline seropositivity data for the study referred to in Bornstein et al, was available from Iversen et al. *** Only baseline seropositivity from the per-protocol population included (PPI) population was presented by specific HPV type (HPV 6/11/16/18/31/33/45/52/58. To be included in the PPI population, individuals had to be seronegative via cLIA assay on day 1 (seropositivity cut offs : ≥20 mMU/mL for HPV16 and ≥24 mMU/mL for HPV18). Therefore, all individuals in this population were cLIA negative at baseline, such that this is likely to represent an underestimate of baseline seropositivity to HPV compared to the wider populations. The original study by Van Damme et al reported that at enrolment, seropositivity to any of the nine HPV types via cLIA assay was detected in 8.5% (163 of 1920) and 5.6% (37 of 666) of girls and boys (aged 9 to 15 years) respectively^*.*

References:

1. Dobson SR, McNeil S, Dionne M, Dawar M, Ogilvie G, Krajden M, Sauvageau C, Scheifele DW, Kollmann TR, Halperin SA, Langley JM. Immunogenicity of 2 doses of HPV vaccine in younger adolescents vs 3 doses in young women: a randomized clinical trial. Jama. 2013 May 1;309(17):1793-802.
2. Iversen OE, Miranda MJ, Ulied A, Soerdal T, Lazarus E, Chokephaibulkit K, Block SL, Skrivanek A, Azurah AG, Fong SM, Dvorak V. Immunogenicity of the 9-valent HPV vaccine using 2-dose regimens in girls and boys vs a 3-dose regimen in women. Jama. 2016 Dec 13;316(22):2411-21.

**Supplementary Figure 1.** Geometric mean concentration (GMCs) / geometric mean titre (GMTs) of HPV16/18 antibodies at month 7 following a 2-dose schedule, by age at vaccination. (a): GMT/GMC of antibodies against HPV16, (b): GMT/GMC of antibodies against HPV18


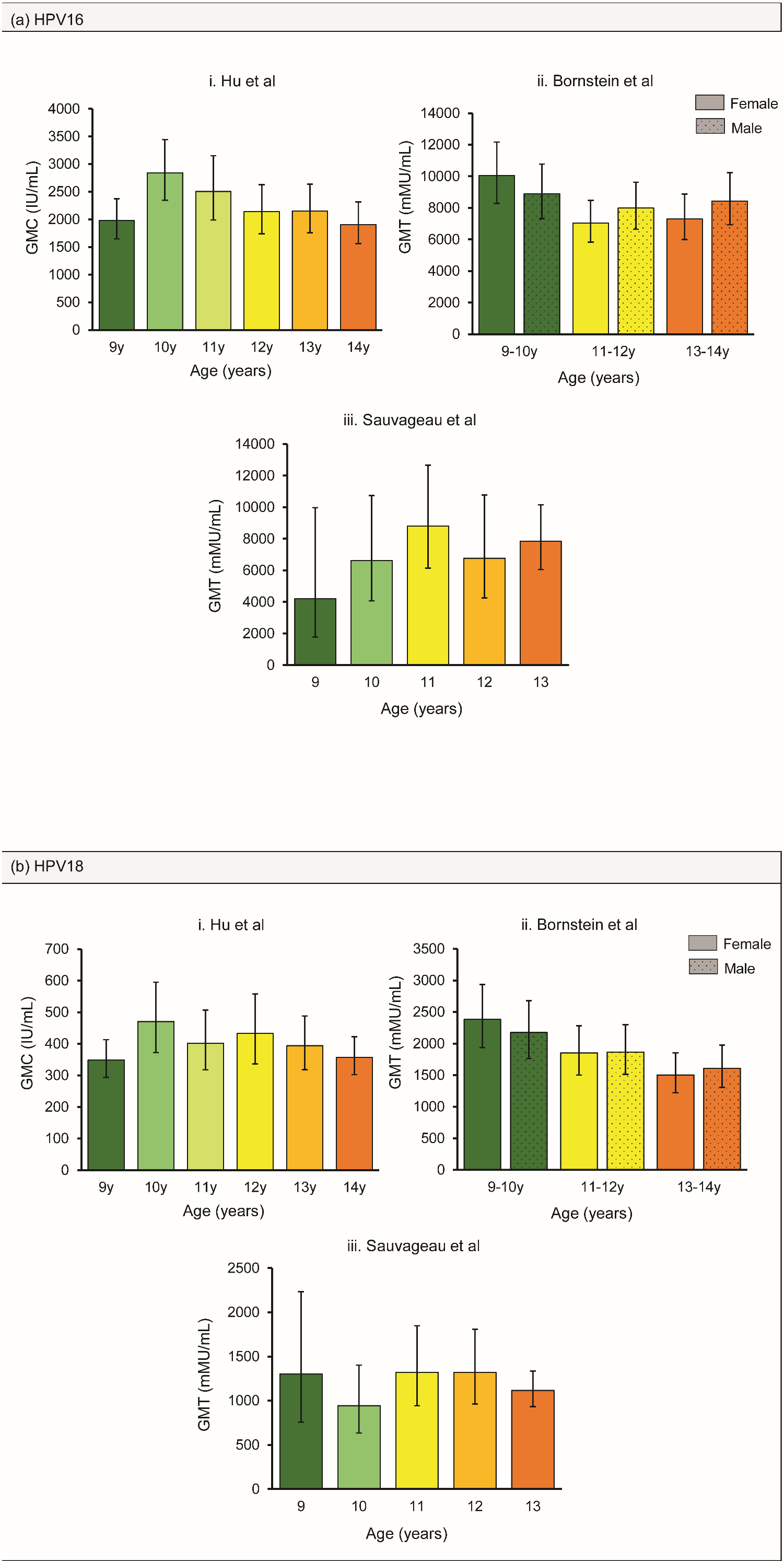


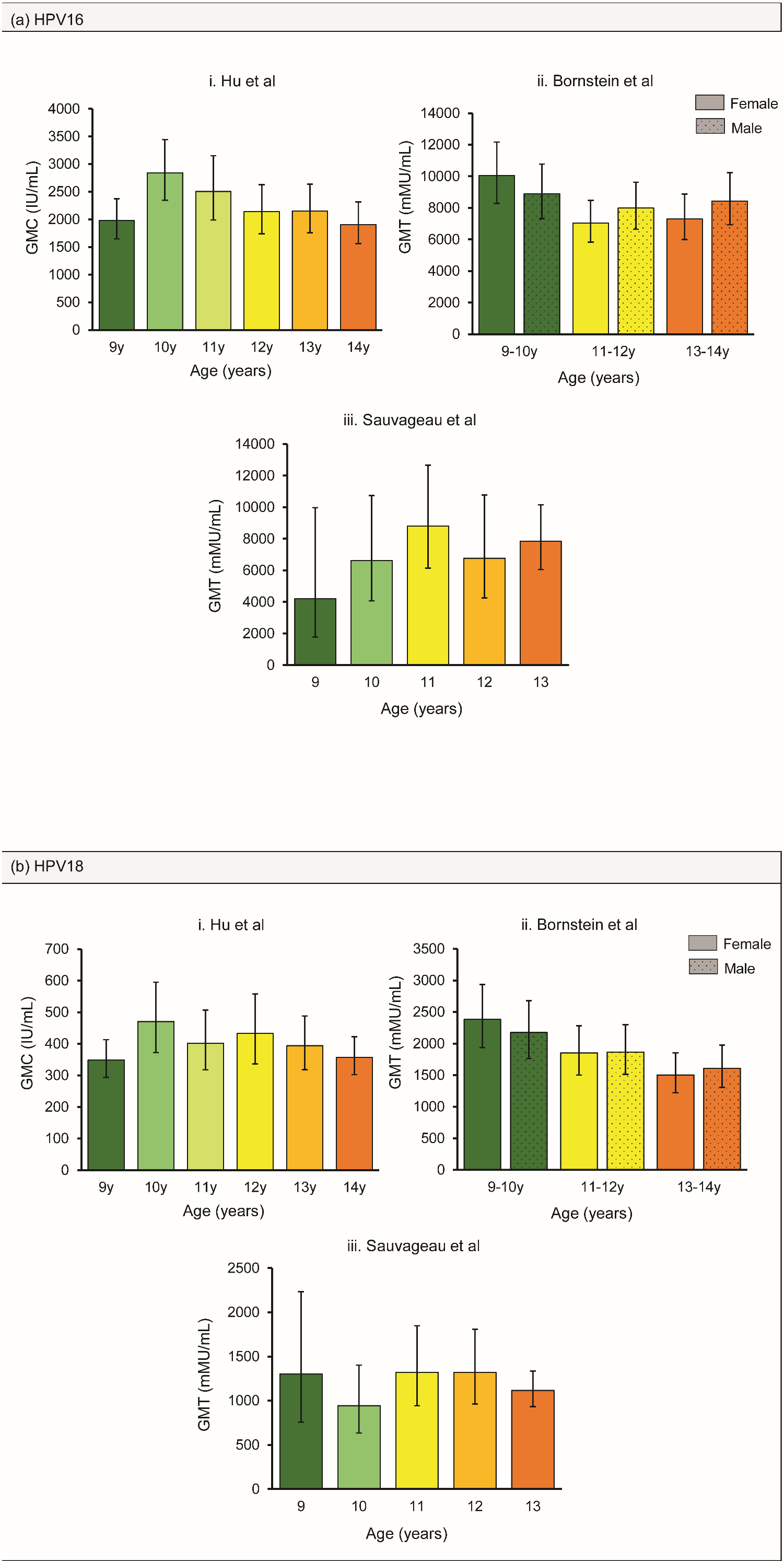


**
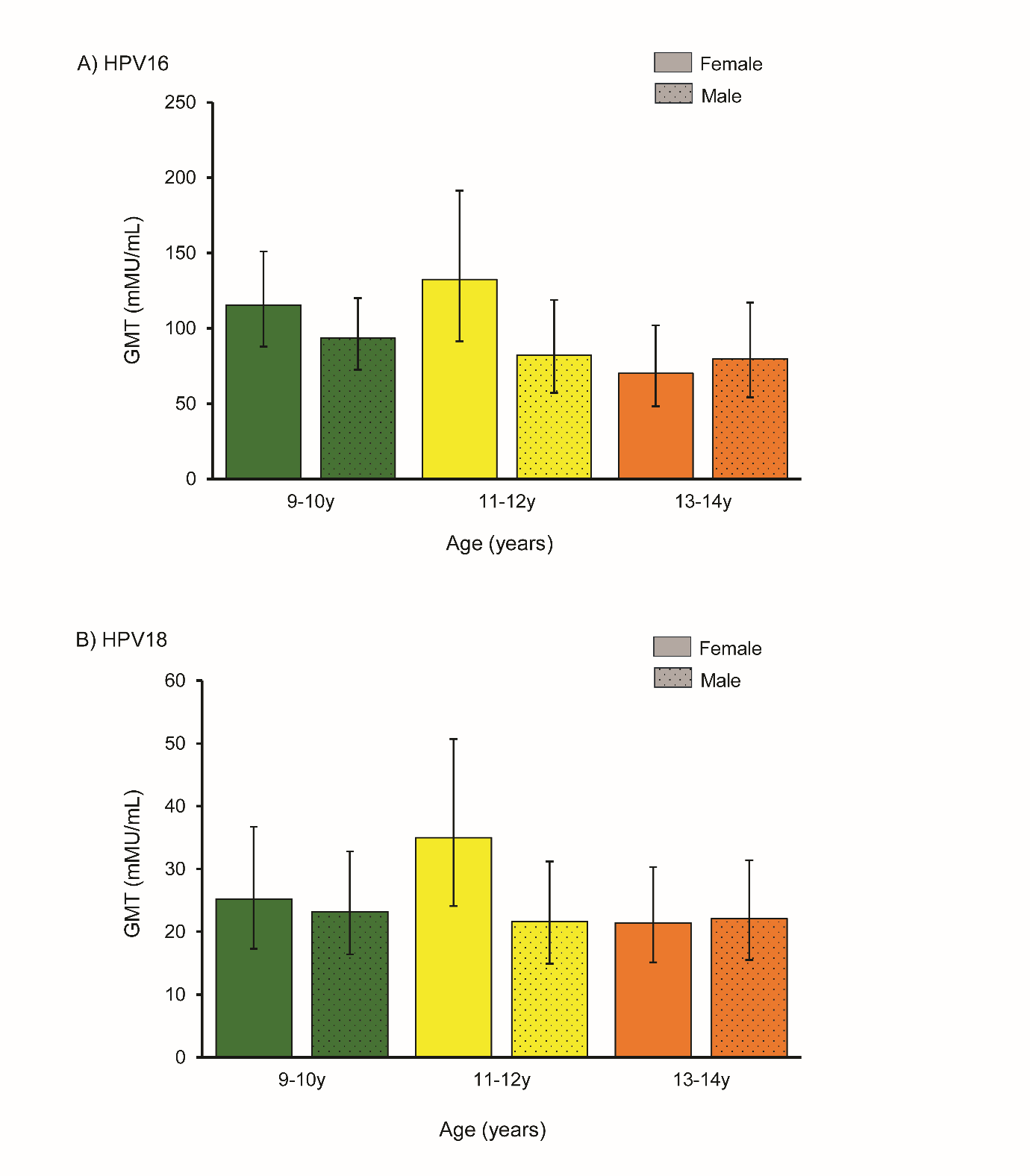
Supplementary Figure 2.** Geometric mean titre of HPV16/18 antibodies at month 12 following a 1-dose schedule, by age at vaccination. (a): GMT of antibodies against HPV16, (b): GMT of antibodies against HPV18

**Supplementary Table 2.** Seropositivity of HPV16/18 antibodies over time, stratified by age at vaccination**.** (a) HPV16 seropositivity. (b) HPV18 seropositivity.

**(a) HPV 16**

|  | | | | | **Seropositivity (%) over time (months)** | | | | | | | | | | | | | | |
| --- | --- | --- | --- | --- | --- | --- | --- | --- | --- | --- | --- | --- | --- | --- | --- | --- | --- | --- | --- |
| **Study** | **Vaccine** | **Dose** | **Age** |  | **M7** | ***N*** | **M12** | ***N*** | **M18** | ***N*** | **M24** | ***N*** | **M30** | ***N*** | **M36** | ***N*** |  | **M126** | ***N*** |
| *Vesikari et al* | 9vHPV | 3 | 9-12y |  | **100** | *137* |  |  |  |  |  |  |  |  |  |  |  |  |  |
|  |  |  | 13-15y |  | **100** | *139* |  |  |  |  |  |  |  |  |  |  |  |  |  |
|  | 4vHPV | 3 | 9-12y |  | **100** | *131* |  |  |  |  |  |  |  |  |  |  |  |  |  |
|  |  |  | 13-15y |  | **100** | *139* |  |  |  |  |  |  |  |  |  |  |  |  |  |
| *Pedersen et al* | 2vHPV | 3 | 9y |  | **100** | *80* |  |  |  |  |  |  |  |  |  |  |  |  |  |
|  |  |  | 10-15y |  | **100** | *153* |  |  |  |  |  |  |  |  |  |  |  |  |  |
| *Reisinger et al* | 4vHPV (boys) | 3 | 9-15y |  | **99.5** | *455* |  |  | **99.3** | *448* |  |  |  |  |  |  |  |  |  |
|  | 4vHPV (girls) | 3 | 9-15y |  | **99.8** | *489* |  |  | **99.8** | *478* |  |  |  |  |  |  |  |  |  |
| *Sauvageau et al* | 4vHPV | 2 | 9-13y |  | **99.5** | *253* |  |  |  |  | **99.5** | *201* |  |  |  |  |  |  |  |
| *Bruce et al* | 4vHPV | 3 | 9-10y |  | **100** | *435** |  |  |  |  | **100** | *351** |  |  |  |  |  |  |  |
|  |  |  | 11-14y |  | **100** | *435** |  |  |  |  | **100** | *351** |  |  |  |  |  |  |  |
| *Hu et al, Yao et al* | 2vHPV | 2 | 9-14y |  | **100** | *267* |  |  |  |  |  |  | **100** | *246* |  |  |  |  |  |
|  |  | 3 | 9-14y |  | **100** | *246* |  |  |  |  |  |  | **100** | *230* |  |  |  |  |  |
| *Bornstein et al* | 9vHPV (girls) | 1 | 9-10y |  |  |  | **95.1** | *39* |  |  |  |  |  |  |  |  |  |  |  |
|  |  |  | 11-12y |  |  |  | **93.6** | *44* |  |  |  |  |  |  |  |  |  |  |  |
|  |  |  | 13-14y |  |  |  | **85.4** | *35* |  |  |  |  |  |  |  |  |  |  |  |
|  | 9vHPV (boys) | 1 | 9-10y |  |  |  | **95.7** | *45* |  |  |  |  |  |  |  |  |  |  |  |
|  |  |  | 11-12y |  |  |  | **85.4** | *41* |  |  |  |  |  |  |  |  |  |  |  |
|  |  |  | 13-14y |  |  |  | **82.1** | *32* |  |  |  |  |  |  |  |  |  |  |  |
|  | 9vHPV (girls) | 2 | 9-10y |  | **100** | *89* |  |  |  |  |  |  |  |  | **97.5** | *78* |  |  |  |
|  |  |  | 11-12y |  | **100** | *89* |  |  |  |  |  |  |  |  | **96.3** | *79* |  |  |  |
|  |  |  | 13-14y |  | **100** | *94* |  |  |  |  |  |  |  |  | **97.7** | *84* |  |  |  |
|  | 9vHPV (boys) | 2 | 9-10y |  | **100** | *88* |  |  |  |  |  |  |  |  | **97.6** | *83* |  |  |  |
|  |  |  | 11-12y |  | **100** | *90* |  |  |  |  |  |  |  |  | **100** | *88* |  |  |  |
|  |  |  | 13-14y |  | **100** | *96* |  |  |  |  |  |  |  |  | **97.8** | *88* |  |  |  |
| *Restrepo et al* | 9vHPV | 3 (cLIA assay) | 9-12y |  | **100** | *774* |  |  |  |  |  |  |  |  |  |  |  | **98.2** | *328* |
|  |  |  | 13-15y |  | **100** | *369* |  |  |  |  |  |  |  |  |  |  |  | **96.7** | *183* |
|  |  | 3 (IgG-LIA assay) | 9-12y |  | **100** | *359* |  |  |  |  |  |  |  |  |  |  |  | **100** | *333* |
|  |  |  | 13-15y |  | **100** | *167* |  |  |  |  |  |  |  |  |  |  |  | **100** | *191* |

**(b) HPV18**

|  | | | | | **Seropositivity (%) over time (months)** | | | | | | | | | | | | | | |
| --- | --- | --- | --- | --- | --- | --- | --- | --- | --- | --- | --- | --- | --- | --- | --- | --- | --- | --- | --- |
| **Study** | **Vaccine** | **Dose** | **Age** |  | **M7** | ***N*** | **M12** | ***N*** | **M18** | ***N*** | **M24** | ***N*** | **M30** | ***N*** | **M36** | ***N*** |  | **M126** | ***N*** |
| *Vesikari et al* | 9vHPV | 3 | 9-12y |  | **100** | *137* |  |  |  |  |  |  |  |  |  |  |  |  |  |
|  |  |  | 13-15y |  | **100** | *139* |  |  |  |  |  |  |  |  |  |  |  |  |  |
|  | 4vHPV | 3 | 9-12y |  | **100** | *131* |  |  |  |  |  |  |  |  |  |  |  |  |  |
|  |  |  | 13-15y |  | **100** | *139* |  |  |  |  |  |  |  |  |  |  |  |  |  |
| *Pedersen et al* | 2vHPV | 3 | 9y |  | **100** | *80* |  |  |  |  |  |  |  |  |  |  |  |  |  |
|  |  |  | 10-15y |  | **100** | *161* |  |  |  |  |  |  |  |  |  |  |  |  |  |
| *Reisinger et al* | 4vHPV (boys) | 3 | 9-15y |  | **99.8** | *458* |  |  | **92.5** | *451* |  |  |  |  |  |  |  |  |  |
|  | 4vHPV (girls) | 3 | 9-15y |  | **99.6** | *494* |  |  | **91.5** | *483* |  |  |  |  |  |  |  |  |  |
| *Sauvageau et al* | 4vHPV | 2 | 9-13y |  | **99.5** | *253* |  |  |  |  | **89** | *201* |  |  |  |  |  |  |  |
| *Bruce et al* | 4vHPV | 3 | 9-10y |  | **100** | *435** |  |  |  |  | **100** | *351** |  |  |  |  |  |  |  |
|  |  |  | 11-14y |  | **100** | *435** |  |  |  |  | **100** | *351** |  |  |  |  |  |  |  |
| *Hu et al, Yao et al* | 2vHPV | 2 | 9-14y |  | **100** | *275* |  |  |  |  |  |  | **99.6** | 253 |  |  |  |  |  |
|  |  | 3 | 9-14y |  | **100** | *262* |  |  |  |  |  |  | **100** | 245 |  |  |  |  |  |
| *Bornstein et al* | 9vHPV (girls) | 1 | 9-10y |  |  |  | **57.5** | *23* |  |  |  |  |  |  |  |  |  |  |  |
|  |  |  | 11-12y |  |  |  | **68.1** | *33* |  |  |  |  |  |  |  |  |  |  |  |
|  |  |  | 13-14y |  |  |  | **52.4** | *32* |  |  |  |  |  |  |  |  |  |  |  |
|  | 9vHPV (boys) | 1 | 9-10y |  |  |  | **55.3** | *26* |  |  |  |  |  |  |  |  |  |  |  |
|  |  |  | 11-12y |  |  |  | **47.9** | *23* |  |  |  |  |  |  |  |  |  |  |  |
|  |  |  | 13-14y |  |  |  | **58.5** | *24* |  |  |  |  |  |  |  |  |  |  |  |
|  | 9vHPV | 2 | 9-10y |  | **100** | *89* |  |  |  |  |  |  |  |  | **97.5** | *78* |  |  |  |
|  |  |  | 11-12y |  | **100** | *89* |  |  |  |  |  |  |  |  | **92.7** | *76* |  |  |  |
|  |  |  | 13-14y |  | **100** | *94* |  |  |  |  |  |  |  |  | **96.5** | *83* |  |  |  |
|  | 9vHPV  (boys) | 2 | 9-10y |  | **100** | *88* |  |  |  |  |  |  |  |  | **97.6** | *83* |  |  |  |
|  |  |  | 11-12y |  | **100** | *96* |  |  |  |  |  |  |  |  | **100** | *88* |  |  |  |
|  |  |  | 13-14y |  | **100** | *88* |  |  |  |  |  |  |  |  | **95.3** | *81* |  |  |  |
| *Restrepo et al* | 9vHPV | 3 (cLIA assay) | 9-12y |  | **100** | *777* |  |  |  |  |  |  |  |  |  |  |  | **84.4** | 333 |
|  |  |  | 13-15y |  | **100** | *372* |  |  |  |  |  |  |  |  |  |  |  | **76** | 192 |
|  |  | 3 (IgG-LIA assay) | 9-12y |  | **99.7** | *359* |  |  |  |  |  |  |  |  |  |  |  | **99.1** | 329 |
|  |  |  | 13-15y |  | **100** | *191* |  |  |  |  |  |  |  |  |  |  |  | **95.6** | 180 |

^*^ *^Bruce et al did not provide the number of participants disaggregated by age group, therefore, the total number of participants was used instead.^*

**Supplementary Table 3.** Table showing the youngest targeted age of HPV vaccination by country/region and income classification.

| Youngest targeted age of vaccination | Countries/regions | | Income Classification (n) | | | Countries |
| --- | --- | --- | --- | --- | --- | --- |
|  | n | % | HIC | MIC | LIC |  |
|  |  |  |  |  |  |  |
| 9 | 46 | 29.9 | 5 | 32 | 9 | Angola, Antigua and Barbuda, Austria, Bahamas, Botswana, Brazil, Burkina Faso, Burundi*, Cambodia, Cameroon, Chile, Colombia, Cook Islands, Côte d'Ivoire, Cuba*, Dominican Republic, Ecuador, El Salvador, Equatorial Guinea**, Eritrea**, Eswatini, Gambia, Grenada, India*, Lesotho, Liberia, Malawi, Mauritania, Mauritius, Montenegro, Mozambique, Myanmar, Namibia*, Nepal*, Nigeria, Papua New Guinea*, Philippines, Senegal, Serbia, Sierra Leone, Solomon Islands, South Africa, Togo, Turkmenistan, Uzbekistan, Vanuatu |
| 10 | 28 | 18.2 | 8 | 18 | 2 | Bangladesh***,Barbados, Belize, Bolivia, Cabo Verde, Costa Rica, Finland, Georgia, Guatemala, Guyana, Kenya, Laos, Maldives, Mali, Micronesia, Moldova, Netherlands, Nicaragua, Panama, Paraguay, Peru, Portugal, Seychelles, Sri Lanka, Suriname, Sweden, Uganda, Zimbabwe. |
| 11 | 33 | 21.4 | 17 | 16 | 0 | Argentina, Bosnia and Herzegovina (Canton sarajevo),Bosnia and Herzegovina (Republika srspska)$, Dominica, France, Greece, Honduras, Indonesia, Italy, Jamaica, Kazakhstan*, Kyrgyzstan,Liechtenstein,Lithuania, Luxembourg, Marshall Islands, Mexico, Monaco, Mongolia, Morocco, New Zealand, Palau, Qatar, Romania, Saint Kitts and Nevis, Saint Lucia, Saint Vincent and the Grenadines, San Marino, Slovenia,Switzerland, Thailand,Trinidad and Tobago, United States of America. |
| 12 | 29 | 18.8 | 21 | 7 | 1 | Andorra,Australia,Bahrain*,Bhutan,Bulgaria, Canada, Cyprus, Denmark,Estonia, Hungary, Iceland, Ireland, Korea (South), Kosovo, Latvia, Libya, Malta, Niue, North Macedonia, Norway, Poland,Rwanda, Singapore, Slovakia, Spain, Tunisia*, United Kingdom, Uruguay Vietnam* |
| 13 | 13 | 8.4 | 7 | 6 | 0 | Albania, Belgium, Bosnia and Herzegovina (FBiH), Brunei, China, Czechia (Czech Republic),Fiji, Germany, Israel, Japan, Malaysia, Samoa, United Arab Emirates, |
| 14 | 5 | 3.3 | 1 | 3 | 1 | Armenia, Croatia, Ethiopia, Tanzania, Zambia |

^*Bahrain, Burundi, Cuba, India, Kazakhstan, Papua New Guinea, Namibia, Nepal, Tunisia, and Vietnam are planned programmes and have not yet been implemented. **Eritrea, Equatorial Guinea and China are pilot programmes. ***Bangladesh: GAVI programme ended in 2024. $ Bosnia and Herzegovina vaccinate by region; therefore, the three regions have been classified separately.^

**Supplementary Table 4**. Table showing the targeted age/age group for HPV vaccination by country/region, income classification and year of programme introduction.

| **Income** | **Country** | **Introduction year** | **Youngest Age targeted** | **Oldest age targeted (if MAC)** |
| --- | --- | --- | --- | --- |
| High income | France | 2006 | 11 | 14 |
| High income | Monaco | 2006 | 11 |  |
| High income | Switzerland | 2006 | 11 | 14 |
| High income | United States of America | 2006 | 11 | 12 |
| High income | Australia | 2007 | 12 | 13 |
| High income | Denmark | 2007 | 12 |  |
| High income | Germany | 2007 | 13 |  |
| High income | Spain | 2007 | 12 |  |
| High income | Greece | 2008 | 11 | 12 |
| High income | Luxembourg | 2008 | 11 | 13 |
| High income | Palau | 2008 | 11 | 12 |
| High income | Panama | 2008 | 10 |  |
| High income | Portugal | 2008 | 10 |  |
| High income | San Marino | 2008 | 11 | 14 |
| High income | United Kingdom | 2008 | 12 | 13 |
| Lower middle income | Bhutan | 2009 | 12 |  |
| High income | Canada | 2009 | 12 |  |
| High income | Italy | 2009 | 11 |  |
| Upper middle income | Marshall Islands | 2009 | 11 |  |
| High income | New Zealand | 2009 | 11 |  |
| Upper middle income | North Macedonia | 2009 | 12 |  |
| High income | Norway | 2009 | 12 |  |
| High income | Slovenia | 2009 | 11 |  |
| High income | Ireland | 2010 | 12 |  |
| High income | Israel | 2010 | 13 |  |
| High income | Latvia | 2010 | 12 |  |
| Upper middle income | Malaysia | 2010 | 13 |  |
| Lower middle income | Micronesia | 2010 | 10 |  |
| High income | Netherlands | 2010 | 10 |  |
| High income | Singapore | 2010 | 12 | 13 |
| High income | Sweden | 2010 | 10 | 11 |
| Upper middle income | Argentina | 2011 | 11 |  |
| High income | Belgium | 2011 | 13 |  |
| High income | Cook Islands | 2011 | 9 |  |
| High income | Iceland | 2011 | 12 |  |
| High income | Japan | 2011 | 13 |  |
| Low income | Rwanda | 2011 | 12 |  |
| High income | Brunei | 2012 | 13 |  |
| Upper middle income | Colombia | 2012 | 9 | 13 |
| High income | Czechia (Czech Republic) | 2012 | 13 |  |
| Upper middle income | Mexico | 2012 | 11 |  |
| High income | Barbados | 2013 | 10 | 11 |
| Upper middle income | Fiji | 2013 | 13 | 14 |
| High income | Finland | 2013 | 10 | 12 |
| Upper middle income | Libya | 2013 | 12 |  |
| High income | Liechtenstein | 2013 | 11 | 14 |
| High income | Malta | 2013 | 12 |  |
| Upper middle income | Paraguay | 2013 | 10 |  |
| Upper middle income | Suriname | 2013 | 10 | 12 |
| High income | Trinidad and Tobago | 2013 | 11 | 12 |
| High income | Uruguay | 2013 | 12 |  |
| High income | Andorra | 2014 | 12 |  |
| High income | Austria | 2014 | 9 |  |
| Upper middle income | Brazil | 2014 | 9 | 13 |
| High income | Chile | 2014 | 9 |  |
| Upper middle income | Ecuador | 2014 | 9 |  |
| High income | Hungary | 2014 | 12 |  |
| High income | Seychelles | 2014 | 10 |  |
| Upper middle income | South Africa | 2014 | 9 |  |
| High income | Bahamas | 2015 | 9 |  |
| Upper middle income | Botswana | 2015 | 9 |  |
| Upper middle income | Peru | 2015 | 10 |  |
| Low income | Uganda | 2015 | 10 |  |
| Lower middle income | Bangladesh | 2016 | 10 |  |
| Upper middle income | Belize | 2016 | 10 |  |
| High income | Croatia | 2016 | 14 |  |
| High income | Cyprus | 2016 | 12 | 13 |
| Lower middle income | Honduras | 2016 | 11 |  |
| High income | Korea (South) | 2016 | 12 |  |
| High income | Lithuania | 2016 | 11 |  |
| Upper middle income | Mauritius | 2016 | 9 |  |
| Lower middle income | Philippines | 2016 | 9 | 14 |
| Upper middle income | Turkmenistan | 2016 | 9 |  |
| Upper middle income | Armenia | 2017 | 14 |  |
| Lower middle income | Bolivia | 2017 | 10 |  |
| High income | Bulgaria | 2017 | 12 | 13 |
| Upper middle income | Dominican Republic | 2017 | 9 | 10 |
| High income | Guyana | 2017 | 10 |  |
| Upper middle income | Jamaica | 2017 | 11 | 12 |
| Upper middle income | Saint Vincent and the Grenadines | 2017 | 11 | 12 |
| Upper middle income | Serbia | 2017 | 9 | 19 |
| Lower middle income | Sri Lanka | 2017 | 10 |  |
| Upper middle income | Thailand | 2017 | 11 |  |
| High income | Antigua and Barbuda | 2018 | 9 | 13 |
| High income | Estonia | 2018 | 12 | 14 |
| Low income | Ethiopia | 2018 | 14 |  |
| Upper middle income | Guatemala | 2018 | 10 | 15 |
| Upper middle income | Indonesia | 2018 | 11 |  |
| Lower middle income | Senegal | 2018 | 9 |  |
| Lower middle income | Tanzania | 2018 | 14 |  |
| Lower middle income | Zimbabwe | 2018 | 10 |  |
| Upper middle income | Costa Rica | 2019 | 10 |  |
| Lower middle income | Cote Divoire | 2019 | 9 |  |
| Upper middle income | Dominica | 2019 | 11 | 12 |
| Low income | Gambia | 2019 | 9 |  |
| Upper middle income | Georgia | 2019 | 10 | 12 |
| Upper middle income | Grenada | 2019 | 9 |  |
| Lower middle income | Kenya | 2019 | 10 |  |
| Low income | Liberia | 2019 | 9 |  |
| Low income | Malawi | 2019 | 9 |  |
| Upper middle income | Maldives | 2019 | 10 |  |
| Upper middle income | Moldova | 2019 | 10 |  |
| High income | Saint Kitts and Nevis | 2019 | 11 | 12 |
| Upper middle income | Saint Lucia | 2019 | 11 |  |
| Lower middle income | Solomon Islands | 2019 | 9 |  |
| High income | United Arab Emirates | 2019 | 13 |  |
| Lower middle income | Uzbekistan | 2019 | 9 |  |
| Lower middle income | Zambia | 2019 | 14 |  |
| Upper middle income | Niue | 2019 | 12 |  |
| Lower middle income | Cameroon | 2020 | 9 |  |
| Upper middle income | El Salvador | 2020 | 9 |  |
| Lower middle income | Myanmar | 2020 | 9 |  |
| High income | Qatar | 2020 | 11 | 26 |
| Lower middle income | Cabo Verde | 2021 | 10 |  |
| Lower middle income | Mauritania | 2021 | 9 |  |
| Low income | Mozambique | 2021 | 9 |  |
| Lower middle income | Sao Tome and Principe | 2021 | N/A |  |
| High income | Slovakia | 2021 | 12 |  |
| Upper middle income | Tuvalu | 2021 | N/A |  |
| Upper middle income | Albania | 2022 | 13 |  |
| Upper middle income | Bosnia and Herzegovina Canton sarajevo | 2022 | 11 | 12 |
| Low income | Burkina Faso | 2022 | 9 |  |
| Upper middle income | China | 2022 | 13 | 15 |
| Low income | Eritrea | 2022 | 9 | 14 |
| Lower middle income | Kyrgyzstan | 2022 | 11 |  |
| Lower middle income | Lesotho | 2022 | 9 |  |
| Upper middle income | Montenegro | 2022 | 9 |  |
| Lower middle income | Morocco | 2022 | 11 |  |
| Lower middle income | Samoa | 2022 | 13 | 14 |
| Low income | Sierra Leone | 2022 | 9 |  |
| Upper middle income | Tonga | 2022 | N/A |  |
| Lower middle income | Vanuatu | 2022 | 9 | 13 |
| Upper middle income | Bosnia and Herzegovina FBiH | 2023 | 13 | 14 |
| Upper middle income | Bosnia and Herzegovina Reupublika srpska | 2023 | 11 | 14 |
| Lower middle income | Cambodia | 2023 | 9 |  |
| Lower middle income | Eswatini | 2023 | 9 |  |
| Lower middle income | Nigeria | 2023 | 9 |  |
| High income | Poland | 2023 | 12 | 13 |
| High income | Romania | 2023 | 11 | 18 |
| High income | Bahrain | Planned for 2023 | 12 | 13 |
| Low income | Togo | 2023 | 9 | 14 |
| Lower middle income | Angola | 2024 | 9 | 12 |
| Upper middle income | Equatorial Guinea | 2024 | 9 | 13 |
| Upper middle income | Kazakhstan | Planned for 2024 | 11 |  |
| Upper middle income | Kosovo | 2024 | 12 |  |
| Lower middle income | Laos | 2024 | 10 |  |
| Low income | Mali | 2024 | 10 |  |
| Upper middle income | Mongolia | 2024 | 11 |  |
| Lower middle income | Nicaragua | 2024 | 10 |  |
| Lower middle income | Papua New Guinea | 2024 | 9 | 14 |
| Low income | Burundi | 2025 | 9 | 13 |
| Upper middle income | Cuba | 2025 | 9 |  |
| Lower middle income | Tunisia | 2025 | 12 |  |
| Lower middle income | Vietnam | 2026 | 12 |  |
| Lower middle income | India | Planned | 9 |  |
| Upper middle income | Namibia | Planned | 9 | 14 |
| Lower middle income | Nepal | Planned | 9 | 14 |

**Supplementary Table 5.** Table of references for Figure 3, Supplementary Table 3 and Supplementary Table 4.

All vaccination programme target ages and years of HPV vaccine introduction were obtained from the

1. WHO [*World Health Organization. Cervical cancer country profiles. Geneva: World Health Organization; 2021 Nov 17. [accessed 2 Jan 2025] Available from:* [*https://www.who.int/publications/m/item/cervical-cancer-country-profiles*](https://www.who.int/publications/m/item/cervical-cancer-country-profiles)*.]*
2. *[World Health Organization. Vaccination schedule for human papilloma virus [Internet]. Geneva: World Health Organization; c2024 [cited 2025 Apr 15]. Available from:*[*https://immunizationdata.who.int/global/wiise-detail-page/vaccination-schedule-for-human-papilloma-virus*](https://immunizationdata.who.int/global/wiise-detail-page/vaccination-schedule-for-human-papilloma-virus)*]*

*except for the below:*

| *Albania* | *https://www.sciencedirect.com/science/article/pii/S2590136224001670#bb0085* |
| --- | --- |
| *Angola* | *https://www.afro.who.int/countries/angola/news/angola-receives-vaccines-fight-cervical-cancer#:~:text=More%20than%201%2C400%2C000%20doses%20of,and%20protection%20against%20cervical%20cancer.* |
| *Australia* | *https://immunisationhandbook.health.gov.au/contents/vaccine-preventable-diseases/human-papillomavirus-hpv* |
| *Bahrain* | *https://www.moh.gov.bh/Content/Files/HealthInfo/Immunization%20summary%20guide%20booklet-23.pdf* |
| *Bangladesh* | *https://www.thelancet.com/journals/lanonc/article/PIIS1470-2045(24)00638-7/fulltext?rss=yes* |
| *Belgium, Denmark* | *https://pmc.ncbi.nlm.nih.gov/articles/PMC8728487/* |
| *Bhutan* | *https://www.exemplars.health/stories/rwanda-and-bhutan-on-course-to-stamp-out-cervical-cancer* |
| *Bosnia and Herzegovina [Canton sarajevo, FBiH,Reupublika srpska]* | *https://pmc.ncbi.nlm.nih.gov/articles/PMC10909086/* |
| *Botswana* | *https://pmc.ncbi.nlm.nih.gov/articles/PMC9102060/* |
| *Burkina Faso* | *https://www.mdpi.com/2076-393X/12/5/489#:~:text=In%202022%2C%20Burkina%20Faso%20launched,%2Dup%20campaign%20%5B44%5D.* |
| *Burundi* | *https://www.unicef.org/burundi/stories/towards-better-sexual-and-reproductive-health-appropriate-services-teenagers-and-young#:~:text=%E2%80%9CBefore%20the%20training%2C%20I%20was,young%20people%20at%20health%20centers.* |
| *Cabo Verde* | *https://unsdg.un.org/latest/stories/girls-today-women-tomorrow-hpv-vaccine-sets-new-course-women-and-girls-cabo-verde#:~:text=Cabo%20Verde%20has%20recently%20introduced,vaccinated%20during%20this%20first%20phase.* |
| *Cambodia* | *https://www.gavi.org/news/media-room/life-saving-hpv-vaccine-introduced-nationwide-routine-immunisation-cambodia* |
| *China* | *https://english.www.gov.cn/statecouncil/ministries/202201/14/content_WS61e0b1c7c6d09c94e48a39ae.html* |
| *Colombia* | *https://www.hpvworld.com/articles/fact-sheet-colombia-www-hpvcentre-net-human-papillomavirus-and-related-cancers/#:~:text=HPV%20vaccination%20for%20girls%20older,2* |
| *Cuba* | *https://www.linkedimmunisation.org/wp-content/uploads/2024/11/HPV-demand-generation-strategies-workshop_Cuba-poster_ENG-SPA-FR-MON.pdf* |
| *Equatorial Guinea* | *https://www.mcd.org/news/first-ever-pilot-vaccination-campaign-hpv-equatorial-guinea#:~:text=June%2021%2C%202024,in%20the%20Baney%20Health%20District.* |
| *Eritrea* | *https://www.gavi.org/vaccineswork/eritrea-touts-screening-and-hpv-vaccines-drive-down-cervical-cancer-rates* |
| *Eswatani* | *https://www.gavi.org/vaccineswork/eswatini-burdened-doubly-hiv-and-cervical-cancer-targets-safety-next-generation#:~:text=with%20the%20disease.-,Vaccine%20intro%20success,said%20Minister%20of%20Health%20Matsebula.* |
| *Fiji* | *https://www.thelancet.com/journals/lanwpc/article/PIIS2666-6065(23)00116-5/fulltext* |
| *Finland* | *https://pubmed.ncbi.nlm.nih.gov/38172018/#:~:text=Background:%20Human%20papillomavirus%20(HPV),of%20adverse%20effects%20(22%25).* |
| *Gambia* | *https://www.sciencedirect.com/science/article/abs/pii/S0264410X23010630#:~:text=For%20example%2C%20as%20presented%20in,campaign%20mode%20vaccination%20in%20schools.* |
| *Guyana, Rwanda, Senegal, Sri Lanka, Uganda* | *https://pmc.ncbi.nlm.nih.gov/articles/PMC10697825/* |
| *India* | *https://www.hpvworld.com/articles/india-prepares-to-introduce-hpv-vaccine-in-national-immunization-program/* |
| *Kazakhstan* | *https://www.who.int/europe/news-room/19-01-2024-protecting-a-generation--kazakhstan-s-commitment-to-hpv-vaccination* |
| *Kyrgyzstan* | *https://www.who.int/europe/news-room/15-02-2023-kyrgyzstan-joins-european-cervical-cancer-prevention-week#:~:text=HPV%20vaccination%20was%20officially%20introduced,14%20have%20already%20been%20vaccinated.* |
| *Laos* | *https://www.gavi.org/sites/default/files/document/proposal-for-nvs---hpv-and-rota-support-2018--lao-pdrpdf.pdf#page28* |
| *Lesotho* | *https://www.gavi.org/sites/default/files/document/2022/Proposal-for-HPV-support-2020-Lesotho.pdf* |
| *Liechtenstein* | *https://vaccine-schedule.ecdc.europa.eu/Scheduler/ByCountry?SelectedCountryId=119&IncludeChildAgeGroup=true&IncludeAdultAgeGroup=true&SelectedVersionId=19* |
| *Luxembourg* | *https://www.sciencedirect.com/science/article/pii/S0264410X18304109* |
| *Mali* | *https://www.gavi.org/vaccineswork/mali-rolls-out-cancer-blocking-jab#:~:text=cervical%20pre%2Dcancer-,Dr%20Diarra%20explained%20why%20the%20programme%20focuses%20on%20ten%2Dyear,Organization's%20recommendation%20to%20prioritise%20the* |
| *Malta* | *https://primaryhealthcare.gov.mt/en/immunisation/* |
| *Mauritania* | *https://www.gavi.org/sites/default/files/document/proposal-for-nvs---hpv-support-2018--mauritaniapdf.pdf#page22* |
| *Mexico* | *https://www.hpvworld.com/articles/mexico-human-papillomavirus-and-related-cancers-fact-sheet-2019/* |
| *Moldova* | *https://pmc.ncbi.nlm.nih.gov/articles/PMC10909086/#:~:text=HPV%20vaccination%20was%20included%20in,Republic%20of%20Moldova%2C%202023).* |
| *Mongolia* | *https://www.linkedimmunisation.org/wp-content/uploads/2024/11/HPV-demand-generation-strategies-workshop-country-poster_Mongolia-ENG-SPA-FR.pdf* |
| *Montenegro* | *https://hpv.srhrpolicyhub.org/country/?region=eu&country=montenegro* |
| *Morocco* | *https://www.hpvworld.com/articles/introduction-of-hpv-vaccination-in-the-kingdom-of-morocco/#:~:text=In%20October%202022%2C%20the%20Moroccan,for%20all%20girls%20aged%2011.* |
| *Mozambique* | *https://www.afro.who.int/countries/mozambique/news/mozambique-boosts-hpv-vaccination-reach-high-coverage#:~:text=In%202018%2C%20WHO%20issued%20a,only%20started%20three%20years%202021.* |
| *Namibia* | *https://www.voanews.com/a/namibia-to-begin-hpv-vaccine-rollout-in-april/7529263.html* |
| *Nepal* | *https://www.who.int/nepal/news/detail/25-11-2024-nepal-holds-high-level-meeting-on-hpv-vaccination-campaign#:~:text=Nepal%20is%20set%20to%20launch,14%20years%20of%20age)%20nationwide.* |
| *Netherlands* | *https://www.vaccinatiecentrum.nl/en/the-hpv-vaccination-are-you-protected/#:~:text=The%20HPV%20vaccination%20according%20to,can%20also%20be%20made%20later.* |
| *Nigeria* | *https://publichealth.jhu.edu/ivac/2024/one-dose-at-a-time-mobilizing-to-eliminate-cervical-cancer-in-nigeria* |
| *North Macedonia* | *https://pmc.ncbi.nlm.nih.gov/articles/PMC10909086/#:~:text=HPV%20vaccination%20was%20included%20in,Republic%20of%20Moldova%2C%202023).* |
| *Papua New Guinea* | *https://www.kirby.unsw.edu.au/news/landmark-vaccination-program-young-girls-papua-new-guinea-now-underway* |
| *Philippines* | *https://www.linkedimmunisation.org/wp-content/uploads/2024/11/HPV-demand-generation-strategies-workshop-poster_Philippines_ENG-SPA-FR-MON.pdf* |
| *Poland* | *https://www.sciencedirect.com/science/article/pii/S2590136224000093* |
| *Romania* | *https://www.unicef.org/romania/stories/young-people-and-hpv-vaccine-awareness-and-challenges#:~:text=In%20Romania%2C%20starting%20from%20December,be%20administered%20by%20family%20doctors.* |
| *Samoa* | *https://www.health.gov.ws/wp-content/uploads/2024/06/MASTER-COPY-CERVICAL-CANCER-ELIMINATION-STRATEGY.pdf* |
| *Serbia* | *https://pmc.ncbi.nlm.nih.gov/articles/PMC10909086/#:~:text=HPV%20vaccination%20was%20included%20in,Republic%20of%20Moldova%2C%202023).* |
| *Sierra Leone* | *https://www.gavi.org/sites/default/files/document/proposal-for-nvs---hpv-routine-and-mr-routine-and-catch-up-campaign-support-2018---sierra-leonepdf.pdf* |
| *Singapore* | *https://www.mims.com/singapore/news-updates/topic/school-based-hpv-vax-scheme-a-success-in-singapore* |
| *Slovakia* | *https://www.frontiersin.org/journals/public-health/articles/10.3389/fpubh.2023.1239963/full* |
| *Suriname* | *https://pmc.ncbi.nlm.nih.gov/articles/PMC9507546/#:~:text=Since%202013%2C%20the%20government%20has,a%20school%2Dbased%20vaccination%20programme.* |
| *Switzerland* | *https://www.mdpi.com/2673-8392/3/2/36* |
| *Togo* | *https://iffim.org/impact/hpv-vaccinations-rebound-2023#:~:text=In%202023%2C%20Gavi%20and%20IFFIm,to%20support%20an%20ongoing%20programme.* |
| *Trinidad and Tobago* | *https://health.gov.tt/sites/default/files/2021-09/HPV%20Vaccine%20-%20Frequently%20Asked%20Questions.pdf* |
| *Tunisia* | *https://www.linkedimmunisation.org/wp-content/uploads/2024/11/HPV-demand-generation-strategies-workshop-country-poster_Tunisia-ENG-SPA-FR.pdf* |
| *Vanuatu* | *https://www.cancercouncil.com.au/news/eliminate-cervical-cancer-png-vanuatu/* |
| *Vietnam* | *https://www.linkedimmunisation.org/wp-content/uploads/2024/11/HPV-demand-generation-strategies-workshop-poster_Vietnam-ENG-SPA-FR-MON.pdf* |
